# DYING WELL WITH DEMENTIA: SUPPORT NEEDS AND DIVERSITY ACROSS CULTURES AND SETTINGS

**DOI:** 10.1093/geroni/igac059.938

**Published:** 2022-12-20

**Authors:** Jenny van der Steen

## Abstract

Dementia is increasingly recognized as a terminal illness but the end of life may comprise an extended period with death difficult to predict. Progressive cognitive impairment negatively affects autonomy. Family caregivers of people with dementia often fulfil important roles as spokespersons, informants, partners in care and proxy decision-makers. However, across cultures and individuals, values around autonomy, being in control, and family involvement are diverse. Therefore, perspectives on end of life with dementia and what is good care during the last phase of life may vary as well. Understanding and measuring good end-of-life care in dementia can help improve person and family centered caregiving at the end of life. This symposium will give insights into what is good end-of-life care in dementia and will show cultural and contextual differences drawing on studies in various countries. The contributions are based on diverse innovative and classical qualitative and quantitative methods including those engaging patient and public. Further work addresses how to support good end-of-life care during the COVID-19 pandemic, given the limitations of family restrictions and the need to provide care virtually. We present research on decision aids, care planning tools and care programs with a focus on ensuring that the perspectives of the person with dementia and family are at the forefront, and address individual differences in perceptions of a good end of life with dementia.

